# Effects of Ferulic Acid Esterase-Producing Lactic Acid Bacteria and Storage Temperature on the Fermentation Quality, In Vitro Digestibility and Phenolic Acid Extraction Yields of Sorghum (*Sorghum bicolor* L.) Silage

**DOI:** 10.3390/microorganisms9010114

**Published:** 2021-01-06

**Authors:** Yixiao Xie, Jingui Guo, Wenqi Li, Zhe Wu, Zhu Yu

**Affiliations:** College of Grassland Science and Technology, China Agricultural University, Beijing 100193, China; bs20183040396@cau.edu.cn (Y.X.); guoyuxiao1993@yeah.net (J.G.); lwqnd@cau.edu.cn (W.L.)

**Keywords:** sorghum silage, ferulic acid esterase, *Lactobacillus plantarum*, *Lactobacillus farciminis*, temperature, fermentation quality, in vitro digestibility, phenolic acids

## Abstract

Two lactic acid bacteria (LAB) strains with different ferulic acid esterase (FAE) activities were isolated: *Lactobacillus farciminis* (LF18) and *Lactobacillus plantarum* (LP23). The effects of these strains on the fermentation quality, in vitro digestibility and phenolic acid extraction yields of sorghum (*Sorghum bicolor* L.) silage were studied at 20, 30 and 40 °C. Sorghum was ensiled with no additive (control), LF18 or LP23 for 45 days. At 40 °C, the lactic acid content decreased, whereas the ammonia nitrogen (NH_3_-N) content significantly increased (*p* < 0.05). At all three temperatures, the inoculants significantly improved the lactic acid contents and reduced the NH_3_-N contents (*p* < 0.05). Neither LP23 nor LF18 significantly improved the digestibility of sorghum silages (*p* > 0.05). The LP23 group exhibited higher phenolic acid extraction yields at 30 °C (*p* < 0.05), and the corresponding yields of the LF18 and control groups were improved at 40 °C (*p* < 0.05). FAE-producing LABs might partially ameliorate the negative effects of high temperature and improve the fermentation quality of sorghum silage. The screened FAE-producing LABs could be candidate strains for preserving sorghum silage at high temperature, and some further insights into the relationship between FAE-producing LABs and ensiling temperatures were obtained.

## 1. Introduction

Silage is a product obtained through a fermentation process during which lactic acid bacteria (LAB) convert water-soluble carbohydrates (WSCs) to organic acids under anaerobic conditions. The production of silage is not only an efficient method for preserving animal feed but is also a process used for the biological pretreatment of lignocellulose materials [1]. Sorghum (*Sorghum bicolor* L.) is one of the most suitable plant materials for ensiling due to its high WSC content and low buffer capacity and is a good choice in marginal areas due to its high yield and excellent drought tolerance [2].

The main factor limiting the use of sorghum silage by ruminants is the digestibility of the fiber. Cellulolytic enzymes, including cellulases and xylanases, are superior fermentation activators for improving fiber degradation [3]. However, the preliminary enzymatic degradation of the plant cell wall is limited by the complex cross-linked structure formed by ferulic acid, which also inhibits the digestion of the cell wall by ruminants [4]. Ferulic acid and *p*-coumaric acid are ubiquitous residues in the plant cell wall and account for 80% of the total phenolic acids [5]. Most ferulic acids form ester bond linkages with arabinoxylans in the primary cell walls, and *p*-coumaric acids, which serve as another indicator of lignification, are also linked to syringyl monolignol units of lignin via ester bonds in the secondary cell walls [6]. Esterification prevents the accessibility of depolymerase towards the cell wall. Microorganisms containing particular ferulic acid esterases (FAEs) can remove side chains attached to the main chains in hemicellulose, and this process makes the polysaccharide backbone accessible to main-chain-degrading enzymes [7]. The free forms of ferulic acids and *p*-coumaric acids are released after disruption of their ester bonds by esterases in the plant cell wall, which might have a variety of implications for the livestock diet [6,8].

The vast majority of feruloyl esterases have been found in fungi (e.g., *Aspergillus niger*, *Penicillium funiculosum*, *Neurospora crassa* and *Talaromyces stipitatus*), and different fungi tend to produce diverse types of esterases with a variety of substrate activities [9]. The hydrolysis rate of an esterase from bacteria, such as *Pseudomonas fluorescens*, is significantly lower than that of fungal feruloyl esterases [9]. However, most esterase-producing fungi are strictly aerobic and cannot be grown under ensiling conditions [10]. Moreover, the addition of exogenous feruloyl esterases would increase the silage cost. Therefore, FAE-producing LAB, which can exhibit tolerance to low pH and anaerobic conditions, might play crucial roles as silage inoculants [10]. FAE-producing LAB makes cross-linked polysaccharides more fragile and thus more easily degraded by rumen due to the synergy between esterases and main-chain degrading enzymes [10]. Several research studies have attempted to study the effects of FAE-producing LAB strains on ensiling and fiber degradability, and some of the findings have shown that LAB-treated silage exhibits higher neutral detergent fiber digestibility (NDFD) [11,12,13].

The success of silage making might also depend on the ability of the LAB to adapt to hot weather [14]. In general, a moderate temperature of 20–30 °C is most suitable for silage fermentation [15]. However, sorghum is mostly harvested and ensiled from June to September in China, which is the hottest period of the year. Researchers have reached a consensus that high temperature usually exerts detrimental effects on the ensiling process. High temperature tends to accelerate the aerobic deterioration of silages and thus leads to butyric and alcoholic fermentation [16]. In addition, some early studies have shown that temperature also significantly affects the activity of FAE produced by LAB [10,17].

Lactic acid bacteria can dominate homolactic or heterolactic fermentation. With the homolactic fermentation, only lactate is produced. In the heterolactic mechanism, ethanol, acetate and CO_2_ are also produced in addition to lactic acid [18]. In contrast to the obligately heterofermentative FAE-producing *Lactobacillus buchneri* strains, whose effects on silage fermentation quality and fiber digestibility have been well studied, we selected a facultatively homofermentative FAE-producing LAB strain (*Lactobacillus plantarum* Orla-Jensen 1919) and an obligately homofermenetative FAE-producing LAB strain (*Lactobacillus farciminis* G.Reuter 1983) to evaluate their enzyme activities at different temperatures. The two LAB strains were applied to sorghum silage stored at different temperatures. To our knowledge, the comparison of the effects of FAE-producing *Lactobacillus farciminis* strains and *Lactobacillus plantarum* strains on ensiling has not been previously documented, and few related studies have directly evaluated the cleaving effects on ester bonds in silage. Therefore, as an indicator of the level of disruption of ester bonds, the ester-linked phenolic acids were also further released and determined in this study.

The objective of this study was to evaluate the effects of isolated FAE-producing LAB and storage temperature on the fermentation quality, in vitro digestibility and phenolic acid extraction yields of sorghum silage.

## 2. Materials and Methods

### 2.1. FAE-Producing LAB Strains

FAE-producing strains were screened from a total of 120 LAB strains using the method described by Donaghy et al. [19]. These strains were isolated from oat silage obtained from grassland at Arhorchin Banner, Inner Mongolia, China (120°05′ E, 43°97′ N, elevation 370 m). Two strains (LP23 and LF18) were confirmed to exhibit outstanding performance in producing FAEs. The LAB strains were preserved in glycerol-containing de Man, Rogosa, Sharpe (MRS) broth at −80 °C.

### 2.2. 16S rRNA Gene Sequencing Analysis

The 16S rRNA gene sequencing analysis was performed using the method described by Xu et al. [10] without modification. The genomic DNA of the two LAB strains was obtained using a bacterial genomic DNA extraction kit (Tiangen, Beijing, China). The DNAs were used as templates for amplification of the 16S rRNA genes by polymerase chain reaction (PCR). Species-specific primers for the amplification of the 16S rRNA genes of *Lactobacillus farciminis* [10] and *Lactobacillus plantarum* [20] were previously reported. The nucleotide sequences were determined by Majorbio Bio-pharm Technology Co., Ltd. (Shanghai, China). Database searches were performed via the usage of the latest release of the non-redundant DNA sequence database present at the NCBI website. DNA sequence similarity searches of the NCBI were performed using the BlastN option of the BLAST program.

### 2.3. Enzymatic Activity Assay

To analyze the enzymatic activity of the two LAB strains, LP23 and LF18 were grown for 18 h in 3 mL of LBZ broth (15 g L^−1^ peptone, 10 g L^−1^ yeast extract, 0.2 g L^−1^ MgSO_4_·7H_2_O, 0.056 g L^−1^ MnSO_4_·H_2_O and 0.26 g L^−1^ K_2_HPO_4_·3H_2_O), which can maintain a neutral environment. The enzymatic assay was conducted in an aerobic environment. The culture was centrifuged at 4000× *g* and 4 °C for 10 min, and the supernatant was collected as the crude enzyme solution of FAE. Mixtures of 400 μL of the crude enzyme solution and 800 μL of a standard substrate solution of methyl ferulate (100 μmol L^−1^) were incubated at 20, 30 and 40 °C for 30 min. The remaining methyl ferulate contents were detected spectrophotometrically at a wavelength of 340 nm. The enzymatic activity of FAE was calculated according to the formula reported by Yue et al. [21].

### 2.4. Sorghum Ensiling

The test was performed from September to November 2018. The sorghum variety (BMR) used were bred by the Hebei academy of agriculture and forestry. Sorghum was grown at the Hengshui Experimental Station of Hebei Academy of Agricultural Sciences, Hebei, China (115°42′ E, 37°53′ N, elevation 22 m), and harvested on the heading date. The test was designed with two LAB inoculants (LP23 and LF18, 1 × 10^6^ cfu g^−1^ fresh sorghum). The chopped sorghum was sprayed with the inoculants using a spray bottle, and the resulting mixtures were mixed thoroughly. In contrast, the control group was sprayed with the same volume of distilled water. The mixtures were packed into 100 mL centrifugal tubes to a density of 750 g L^−1^ (fresh matter). Nine tubes of each inoculant were prepared, and three tubes of each treatment were stored in incubators at different temperatures (20, 30 and 40 °C). All the tubes were opened and sampled after ensiling for 45 days.

### 2.5. Chemical Analysis

After opening, the content of each tube was removed and blended thoroughly. Twenty grams of the sorghum silage samples were mixed with 180 mL of distilled water and homogenized for 5 min in a blender jar. The mixture was filtered, and the filtrate was centrifuged at 10,000× *g* and 4 °C for 5 min. The supernatant was used for determination of the pH value and the organic acid and ammonia nitrogen (NH_3_-N) contents. The pH value was measured using an electrode (PHS-3C, INESA, Shanghai, China). The contents of lactic, acetic, propionic and butyric acids were analyzed using a high-performance liquid chromatography (HPLC) instrument (Shimadzu-10A, Kyoto, Japan) equipped with a Shodex Rs Pak KC-811 S-DVB gel column (8.0 mm × 30 cm; Shimadzu, Kyoto, Japan) based on the procedure described by Tian et al. [22]. The NH_3_-N content was analyzed using the sodium hypochlorite and phenol method [23]. To assess the quality of sorghum silage, Flieg’s point was calculated based on the dry matter (DM) content and pH value of the sorghum silage [24].

The DM content was determined by oven-drying at 65 °C for 48 h. The dried samples were milled and passed through a 1.0 mm screen and then used to analyze the WSC, crude protein (CP), amylase-treated neutral detergent fiber (aNDF), acid detergent fiber (ADF) and acid detergent lignin (ADL) contents, as described by Tian et al. [22]. Both aNDF and ADF are expressed inclusive of residual ash. The hemicellulose and cellulose contents were estimated as the aNDF values minus the ADF values and the ADF values minus the ADL values, respectively. The ash content was determined by ignition at 550 °C. The weight loss was calculated using fresh weight of sorghum measured before and after ensiling. The DM loss was corrected for the DM content of fresh forage and its respective silage [25].

### 2.6. In Vitro Incubation and Degradability Measurements

Rumen fluid was obtained from four Angus steers before morning feeding. These steers were fed 2.5 kg (DM) of whole-plant corn silage, 1.5 kg of corn stalks, 2.2 kg of corn grain, 0.6 kg of jujube powder, 0.56 kg of soybean meal, 0.32 kg of corn germ oil meal, 0.06 kg of baking soda, 0.06 kg of salt and 0.2 kg of premix. In vitro incubation was performed for 48 h according to the procedure described by Menke et al. [26]. The reduced weights of the samples were used to calculate the in vitro dry matter digestibility (DMD). The aNDF contents of the residues were also analyzed to calculate the in vitro NDFD.

### 2.7. Extraction and Determination of Phenolic Acids

According to the method described by Cao et al. [6], the ester-linked phenolic acids were released and extracted with 2.0 mol L^−1^ anaerobic sodium hydroxide solution (added under a N_2_ stream) at 39 °C for 24 h in the dark. The mixture was acidified to pH value below 2.0 with concentrated phosphoric acid. The standard chemicals of phenolic acids and the phenolic acids extracted from silage were both directly analyzed using an HPLC instrument (Shimadzu -10A, Kyoto, Japan) equipped with a Reprosil-Pur Basic C18 column (4.6 mm × 25 cm; Dr. Maisch, Germany). A 40% methanol solution (*v*/*v*) containing 0.06% formic acid was used as the mobile phase at a flow rate of 0.5 mL min^−1^. The column temperature was 40 °C, and the compounds were detected spectrophotometrically at a wavelength of 320 nm.

### 2.8. Statistical Analysis

The data are expressed as the means ± standard errors of the mean (SEMs). Using SPSS version 19.0 for Windows (SPSS Inc., Chicago, IL, USA), the data were analyzed by one-way or two-way analysis of variance (ANOVA) with the inoculants and ensiling temperature as the fixed effects. The differences among the means obtained with the various treatments were assessed using Duncan’s multiple range method. A value of *p* < 0.05 was considered significant.

## 3. Results

### 3.1. Identification of FAE-Producing LAB Strains and Their Enzymatic Activity

**Figure 1:**
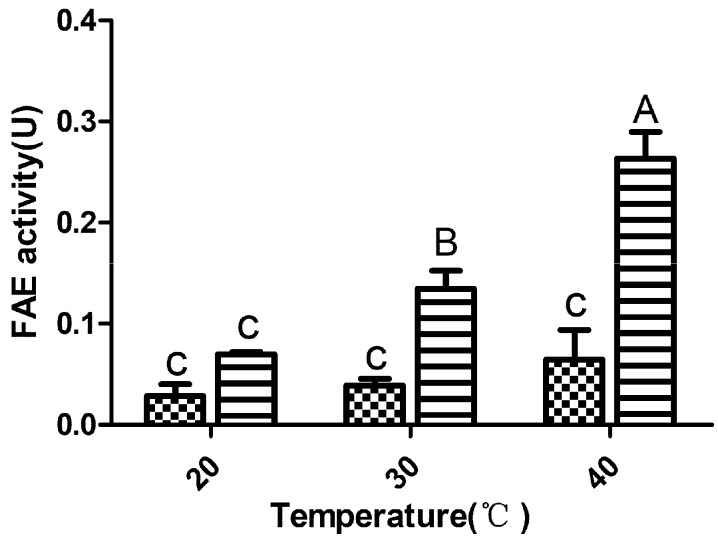
The ferulic acid esterase enzymatic activity. LF18, *Lactobacillus farciminis* inoculant 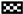; LP23, *Lactobacillus plantarum* inoculant 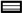; FAE, ferulic acid esterase. Means with difference superscripts (A–C) differ significantly from each other (*p* < 0.05). Error bars indicate standard error of means.

The two LAB strains were identified as *Lactobacillus plantarum* (LP23) and *Lactobacillus farciminis* (LF18) via 16S rRNA sequencing. Figure 1 shows the FAE enzymatic activities of LF18 and LP23 at different temperatures. At 30 and 40 °C, the FAE enzymatic activity of LP23 was higher than that of LF18 (*p* < 0.05).

### 3.2. Chemical Composition and Phenolic Acid Extraction Yields of Sorghum Prior to Ensiling

Before ensiling, the sorghum samples had a DM content of 375.67 g kg^−1^, a WSC content of 70.31 g kg^−1^ DM, a CP content of 102.10 g kg^−1^ DM, an aNDF content of 598.84 g kg^−1^ DM, an ADF content of 343.19 g kg^−1^ DM, a cellulose content of 298.17 g kg^−1^ DM, a hemicellulose content of 255.76 g kg^−1^ DM, an ADL content of 45.02 g kg^−1^ DM, an ash content of 88.55 g kg^−1^ DM, a ferulic acid extraction yield of 1.88 g kg^−1^ DM and a *p*-coumaric acid extraction yield of 5.61 g kg^−1^ DM.

### 3.3. Fermentation Characteristics of Sorghum Silages

The fermentation characteristics of the sorghum silage samples are shown in Table 1. The interaction is the effect produced by the mutual influence of two or more processing factors. In this study, the interactions between the temperature and the inoculant had significant effects on the acetic acid level, the ratio of lactic acid to acetic acid and the NH_3_-N content (*p* < 0.05). The temperature significantly affected all the fermentation characteristics with the exception of the ratio of lactic acid to acetic acid (*p* < 0.05). The inoculants had no significant effect on the ratio of lactic acid to acetic acid, the acetic acid level or the propionic acid level (*p* > 0.05). 

**Figure 2:**
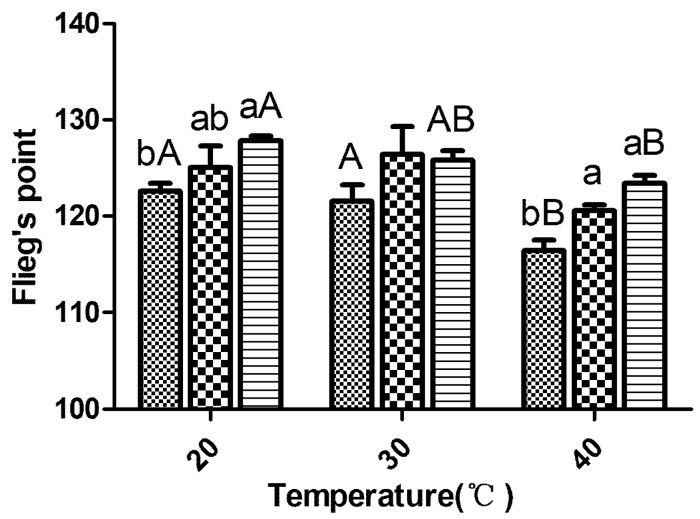
The Flieg’s point of sorghum silage. Control 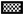; LF18, *Lactobacillus farciminis* inoculant 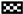; LP23, *Lactobacillus plantarum* inoculant 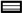. Means within the same temperature (a, b) or within the same inoculant (A, B) with difference superscripts differ significantly from each other (*p* < 0.05). Error bars indicate standard error of means.

The pH values, the propionic acid and NH_3_-N contents of each treatment group tended to be higher with increases in the temperature. The inoculants could lower both the pH value and the NH_3_-N content, and these effects were obtained at the three different temperatures (*p* < 0.05). At 20 and 40 °C, the inoculants increased the lactic acid level and significantly improved Flieg’s point of the silage (*p* < 0.05) (Figure 2). At 20 and 30 °C, the inoculants tended to reduce the acetic acid content in the silage, although the reduction obtained with LP23 was more significant than that obtained with LF18 (*p* < 0.05), which resulted in a higher lactic acid-to-acetic acid ratio in the LP23-treated silages (*p* < 0.05). However, the LP23 group produced the most acetic acid at 40 °C, and no significant difference in the ratio of lactic acid to acetic acid was found among the treatments (*p* > 0.05). At 20 °C, no propionic acid was detected in the silage samples, whereas at 30 °C, the inoculants significantly decreased the propionic acid content (*p* < 0.05). No butyric acid was detected in any of the tested samples.

### 3.4. Chemical Composition of Sorghum Silages

The chemical compositions of the sorghum silage samples are shown in Table 2. No significant interaction effect between temperature and inoculant was found in the chemical composition of the silages (*p* > 0.05). The inoculants significantly affected the WSC, aNDF, hemicellulose and ash contents and the loss of weight (*p* < 0.05). Temperature exerted significant effects on the WSC, CP, aNDF, ADF and ADL contents (*p* < 0.05).

Differences in the chemical composition among the inoculant-treated samples were only found at 20 and 30 °C (*p* < 0.05). The inoculants tended to preserve more WSCs, and the LP23 group had significantly higher WSC levels than the control group at 30 °C (*p* < 0.05). The LF18- and control-treated silage had a higher WSC content at 20 than 30 °C and 40 °C, whereas the LP23-treated silage preserved more WSCs at both 20 and 30 °C (*p* < 0.05). Increases in temperature tended to be associated with reductions in the WSC and CP contents and increases in the aNDF and ADL contents. The LP23- and LF18-treated silages stored at both 30 and 40 °C had a lower CP content than those stored at 20 °C (*p* < 0.05). The inoculants could also reduce the aNDF content at 30 °C (*p* < 0.05). All the silages stored at 40 °C had a higher ADL level (*p* < 0.05), and the LP23-treated silages ensiled at 20 °C had the lowest ADL content (*p* < 0.05). The inoculants decreased the weight losses and increased the ash contents (*p* < 0.05).

### 3.5. In Vitro Incubation and Degradability Measurements

The in vitro digestibility of sorghum silage is shown in Table 3. No significant differences in the DMD were found among the different temperatures and treatments. The interaction of temperature and inoculant on the NDFD was detected (*p* < 0.05). The LP23 group showed a higher NDFD than the other groups at 30 and 40 °C, but the improvement in the NDFD was significant only compared with that of the LF18 group (*p* < 0.05).

### 3.6. Phenolic Acid Extraction Yields of Sorghum Silage

**Figure 3:**
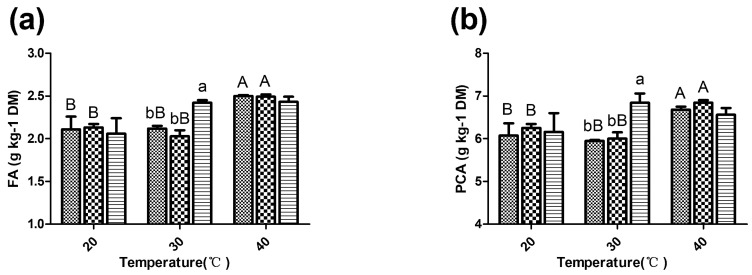
The phenolic acid extraction yields of sorghum silage. (**a**) Ferulic acid (FA) extraction yields. (**b**) *p*-coumaric acid (PCA) extraction yields. DM, dry matter; Control 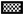; LF18, *Lactobacillus farciminis* inoculant 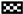; LP23, *Lactobacillus plantarum* inoculant 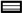. Means within the same temperature (a, b) or within the same inoculant (A, B) with difference superscripts differ significantly from each other (*p* < 0.05). Error bars indicate standard error of means.

The phenolic acid extraction yields are shown in Figure 3. At 30 °C, the LP23 group presented significantly higher phenolic acid extraction yields than the LF18 and control groups (*p* < 0.05). The phenolic acid extraction yields obtained with the control- and LF18-treated silage at 40 °C were greatly improved compared with those at the other temperatures (*p* < 0.05).

## 4. Discussion

All the sorghum silage samples were well fermented with low pH values (<4.2) because the WSC content of the raw material was high (70.31 g kg^−1^ DM), adequate fermentable substrate is the basis for sufficient lactic acid production [27]. However, it was found that high temperature could cause a decline in the lactic acid content and increases the pH value previously [14], which also occurred in our study. The homofermentative inoculants produced a sufficient amount of lactic acid, and the LAB-treated silages in this study all had lower pH values than the control. Filya et al. [28] and Alhaag et al. [29] also reported that the application of *Lactobacillus plantarum* to sorghum silage significantly improved the lactic acid contents and reduced the acetic acid and propionic acid contents. The efficiency of the fermentation was promoted by the LAB treatments at 20 and 30 °C. The inoculants accelerated the process of lactic acid fermentation and suppressed the detrimental microorganisms such as clostridia, enterobacteria and yeasts, which reduced the consumption of WSCs [2]. The silages treated with homolactic acid inoculant usually had a higher ratio of lactic acid to acetic acid, which is usually used as a qualitative indicator of fermentation [30]. The LP23-treated silage had a significantly higher ratio of lactic acid to acetic acid than the control and LF18 groups at 20 and 30 °C, but at a temperature of 40 °C, the ratio of lactic acid to acetic acid in the LP23 group was significantly decreased. It is likely that LP23 does not exhibit its optimal growth at high temperature and becomes less competitive than enterobacteria at the higher temperature used in this study. In contrast to homofermentative LAB, enterobacteria produce more acetic acid from glucose [31]. A lower temperature might reduce the propionic acid content. Wang et al. [32] did not detect propionic acid in silage stored at a temperature below 25 °C. In China, sorghum is typically harvested during the hottest four months, when daytime temperatures can easily reach 40 °C, while tropical regions may maintain high temperatures for longer periods of time. In this study, the LAB-treated silages presented a higher Flieg’s point than the control silages at 40 °C, which indicated that the inoculants could still improve the fermentation quality of sorghum silage at high temperature.

Furthermore, increased proteolysis occurs in silages stored at high temperatures, which results in lower CP contents [33,34]. In addition, increased proteolysis results in higher buffering and poorer fermentation, which might also alter the fermentation profile and the microbiology of sorghum silage [31]. In this study, the CP contents slightly decreased at 30 or 40 °C. The NH_3_-N contents also increased at higher temperature, but the change was extremely small. Nevertheless, regardless of the storage temperature, all LAB-treated sorghum silages had lower NH_3_-N contents than the control silages, which is in agreement with the results reported by Wang et al. [32,35]. This finding might have been obtained because the selected strains do not ferment amino acids and because protein breakdown was reduced due to a rapid decrease in pH [36].

The inoculants performed good in reducing the fresh weight losses of silage but did not reduce the DM losses. This might be due to the higher loss of volatile products caused by the oven drying, which could also explain the higher ash contents in the inoculant-treated silages [37]. The high temperature of 40 °C did not cause significantly higher DM losses in this study. However, the silages stored at high temperature still had higher ADL contents. Thus, we tentatively conclude that the high temperature promoted the Maillard reaction, which increased the Maillard polymer contents in the sorghum silage [38]. The inoculants tended to reduce the aNDF and hemicellulose contents of sorghum silage at 20 and 30 °C. This result confirmed the positive effect of the inoculants on fiber degradation. The LAB-treated silages had lower aNDF contents at 30 °C, but no significant difference was detected at 40 °C. A similar phenomenon observed by Li et al. [39], who inoculated corn stalk silages with FAE-producing LAB and stored the samples at different temperatures. These researchers also found that FAE-producing LAB promoted the degradation of lignocellulosic biomass at lower temperature, but the effect in the absence of cellulase was extremely small [39].

The NDFD of the LP23 group was significantly higher than that of the LF18 group at 30 and 40 °C, which exactly corresponded to the FAE enzymatic activity of the two LAB strains at the same temperatures, and this finding indicates that FAE might potentially improve the NDFD of sorghum silage. However, it should be noted that the anaerobic and acidic conditions might lead to a different FAE enzymatic activity than that found in the broth. The activity of the FAE produced by the inoculants might be inhibited in an actual ensiling environment [17]. Furthermore, small molecule ferulic acid esters (such as methyl or ethyl ferulate) are usually used in screening for enzyme activity. But these substrates are different from the actual plant ferulate, so we also would like to point out that other characteristic substrates may be able to better predict their effect in silage, which is what we plan to investigate in the future.

No significant difference in NDFD was found between the control and LAB-treated silages, which might be due to the epiphytic bacteria in the control group also exhibiting better NDFD-enhancing characteristics than LF18. In the study conducted by Jin et al. [40,41], the improvement in NDFD was found to be more dependent on other characteristics of the inoculants rather than the expression of FAE. However, due to the potential of FAE to improve digestibility, it may be possible in the future to screen for more effective FAE-producing LAB to degrade the fiber and improve digestibility of silage using the more appropriate methods we mentioned above. Furthermore, cell wall digestibility depends on multiple factors, and further research is needed to explore the potential interactions between FAE-producing LAB and NDFD.

The FAE enzymatic activity was low at 20 °C; thus, no significant difference in the phenolic acid extraction yields was found among the three groups. A higher phenolic acid extraction yield was obtained with LP23-treated silage than with the control and LF18-treated groups at 30 °C, which corresponded to the enzymatic activity data. The control and LF18 groups also presented high ferulic acid and *p*-coumaric acid extraction yields at 40 °C; as a result, no significant difference in the phenolic acid extraction yields were found among the three treatments at this temperature, although the LP23 strain used in this study also presented its highest FAE enzymatic activity at 40 °C. The results indicated that high temperature might exert comparable effects on the cleaving of ester bonds to esterase. In fact, the previous analysis of enzyme activity showed that methyl ferulate (the substrate of FAE) in blank medium with no FAE-producing LAB was also greatly degraded at 40 °C. In view of the difference in NDFD between the LP and LF groups at 40 °C, it is possible to infer that high temperature and esterase caused ester bond rupture in different ways. The cleaving effect in the LF group was more dependent on the high temperature, which might have less impact on the digestibility. We speculated that the reduction in aNDF might also rely on the own characteristics of the LABs and that the disruption of ester bonds at high temperature might exert little effect on the aNDF content. Therefore, the sorghum silage stored at 40 °C still had a high content of aNDF, even though the ester bonds might have been notably disrupted. This finding can also partly explain the lack of significant difference in the aNDF content among the groups at high temperatures, as was discussed above. Further studies are needed to determine the connection between the disruption of ester bonds caused by different mechanisms and the characteristics of silage fermentation.

## 5. Conclusions

Although high temperatures exert detrimental effects on the chemical composition and fermentation characteristics of sorghum silage, the inoculation of silage with LAB in this study improved the Flieg’s point at 40 °C, which partially ameliorated the negative effects of high temperature and improved silage quality. The inoculants showed positive effects on fiber degradation and the FAE showed potential improvement on NDFD. However, in this study, the addition of the LP23 and LF18 strains did not effectively improve the digestibility of silage. Both high temperature and FAE exerted effects on the cleavage of ester bonds and improved the phenolic acid extraction yields. The results provide further insights into the relationship between FAE-producing LAB strains and ensiling temperatures.

## Figures and Tables

**Table 1:** Fermentation characteristics of sorghum silages.

Item	Temperature		Inoculant		SEM	Significance		
		Control	LF18	LP23		I	T	I × T
pH	20 °C	3.92 ^aB^	3.81 ^b^	3.72 ^bC^	0.01	<0.001	<0.001	0.705
	30 °C	3.93 ^aB^	3.82 ^b^	3.77 ^bB^				
	40 °C	4.04 ^aA^	3.90 ^b^	3.86 ^bA^				
Lactic acid (g kg^−1^DM)	20 °C	47.9 ^bB^	54.1 ^abAB^	63.5 ^aA^	0.82	0.005	<0.001	0.111
	30 °C	60.7 ^A^	58.6 ^A^	62.4 ^A^				
	40 °C	43.3 ^bC^	44.0 ^abB^	48.1 ^aB^				
Acetic acid (g kg^−1^ DM)	20 °C	14.7 ^aA^	13.6 ^aA^	10.0 ^bB^	0.33	0.326	<0.001	<0.001
	30 °C	14.9 ^aA^	13.8 ^abA^	10.8 ^bAB^				
	40 °C	7.1 ^bB^	8.7 ^abB^	12.4 ^aA^				
Propionic acid (g kg^−1^ DM)	20 °C	ND ^C^	ND	ND ^C^	0.18	0.602	<0.001	0.537
	30 °C	1.3 ^aB^	1.0 ^b^	0.4 ^cB^				
	40 °C	1.9 ^A^	3.2	2.4 ^A^				
Butyric acid (g kg^−1^ DM)	20 °C	ND	ND	ND	-	-	-	-
	30 °C	ND	ND	ND				
	40 °C	ND	ND	ND				
Lactic acid: Acetic acid ratio	20 °C	3.26 ^bB^	4.12 ^b^	6.39 ^aA^	0.20	0.209	0.399	0.002
	30 °C	4.13 ^bAB^	4.25 ^b^	5.89 ^aA^				
	40 °C	6.69 ^A^	5.19	3.89 ^B^				
NH_3_-N (g kg^−1^ TN)	20 °C	22.9 ^aC^	17.7 ^bC^	16.5 ^bC^	0.27	<0.001	<0.001	<0.001
	30 °C	28.8^a B^	21.1 ^bB^	21.9 ^bB^				
	40 °C	40.7 ^aA^	27.3 ^bA^	27.0 ^bA^				

DM, dry matter; NH_3_-N, ammonia nitrogen; TN, total nitrogen; LF18, *Lactobacillus farciminis* inoculant; LP23, *Lactobacillus plantarum* inoculant; ND not detected; I, inoculant; T, temperature; I × T, interaction between inoculant and temperature. Means within the same row (a,b) or within the same column (A–C) with difference superscripts differ significantly from each other (*p* < 0.05). SEM, standard error of means.

**Table 2:** Chemical compositions of sorghum silages.

Item	Temperature		Inoculant		SEM	Significance		
		Control	LF18	LP23		I	T	I × T
DM (g kg^−1^ FM)	20 °C	371.1	362.7	357.9	1.88	0.218	0.729	0.524
	30 °C	369.8	370.2	358.4				
	40 °C	364.9	357.9	364.6				
WSC (g kg^−1^ DM)	20 °C	39.0 ^A^	41.2 ^A^	42.8 ^A^	0.42	0.010	<0.001	0.053
	30 °C	32.0 ^bB^	35.4 ^abB^	38.4 ^aA^				
	40 °C	32.4 ^B^	35.7 ^B^	31.7 ^B^				
CP (g kg^−1^ DM)	20 °C	109.8 ^A^	109.9 ^A^	109.7 ^A^	0.34	0.262	<0.001	0.195
	30 °C	109.4 ^A^	105.6 ^B^	105.9 ^B^				
	40 °C	104.7 ^B^	105.6 ^B^	104.1 ^B^				
aNDF (g kg^−1^ DM)	20 °C	572.9 ^B^	564.6	557.4 ^C^	1.40	0.014	<0.001	0.273
	30 °C	585.2 ^aA^	569.3 ^b^	571.5 ^bB^				
	40 °C	585.2 ^A^	579.2	585.5 ^A^				
ADF (g kg^−1^ DM)	20 °C	314.1	313.0	308.0 ^B^	1.59	0.754	0.023	0.293
	30 °C	319.1	315.5	314.0 ^B^				
	40 °C	315.4	328.0	327.1 ^A^				
HC (g kg^−1^ DM)	20 °C	258.8	251.6	249.5	1.95	0.040	0.347	0.889
	30 °C	266.1	253.8	257.5				
	40 °C	269.8	251.2	258.4				
CE (g kg^−1^ DM)	20 °C	293.5	291.3	288.4	1.78	0.556	0.379	0.232
	30 °C	296.2	294.4	292.3				
	40 °C	277.0	295.4	291.8				
ADL (g kg^−1^ DM)	20 °C	20.6 ^B^	21.7 ^B^	19.5 ^C^	0.58	0.317	<0.001	0.396
	30 °C	26.8 ^B^	25.2 ^B^	25.9 ^B^				
	40 °C	38.4 ^A^	32.6 ^A^	35.3 ^A^				
Ash (g kg^−1^ DM)	20 °C	90.0	93.3 ^A^	91.6	0.33	0.034	0.438	0.564
	30 °C	89.3	90.6 ^B^	92.2				
	40 °C	89.7	91.1 ^B^	91.2				
Weight loss (g kg^−1^ FM)	20 °C	28.9 ^a^	13.8 ^b^	13.6 ^b^	1.05	0.005	0.309	0.106
	30 °C	24.6	14.6	13.1				
	40 °C	14.4	15.5	14.5				
DM loss (g kg^−1^ DM)	20 °C	40.7	47.9	60.3	4.73	0.538	0.788	0.561
	30 °C	40.1	29.1	58.5				
	40 °C	42.6	62.0	43.7				

DM, dry matter; FM, fresh matter; WSC, water-soluble carbohydrate; CP, crude protein; aNDF, amylase-treated neutral detergent fiber; ADF, acid detergent fiber; HC, hemicellulose; CE, cellulose; ADL, acid detergent lignin; LF18, *Lactobacillus farciminis* inoculant; LP23, *Lactobacillus plantarum* inoculant; I, inoculant; T, temperature; I × T, interaction between inoculant and temperature. Means within the same row (a, b) or within the same column (A–C) with difference superscripts differ significantly from each other (*p* < 0.05). SEM, standard error of means.

**Table 3:** In vitro digestibility of sorghum silages.

Item	Temperature		Inoculant		SEM	Significance		
		Control	LF18	LP23		I	T	I × T
DMD (%)	20 °C	47.71	47.42	48.78	0.78	0.866	0.485	0.966
	30 °C	49.1	49.35	49.15				
	40 °C	48.9	51.53	50.5				
NDFD (%)	20 °C	35.61	36.96	34.52	0.30	0.042	0.876	0.018
	30 °C	34.30 ^ab^	33.30 ^b^	38.34 ^a^				
	40 °C	35.59 ^ab^	33.40 ^b^	37.36 ^a^				

DMD, dry matter digestibility; NDFD, neutral detergent fiber digestibility; LF18, *Lactobacillus farciminis* inoculant; LP23, *Lactobacillus plantarum* inoculant; I, inoculant; T, temperature; I × T, interaction between inoculant and temperature. Means within the same row (a, b) with difference superscripts differ significantly from each other (*p* < 0.05). SEM, standard error of means.

## Data Availability

Data is contained within the article.

## References

[1] Tišma M., Planinić M., Bucić-Kojić A., Panjičko M., Zupančič G.D., Zelić B. (2018). Corn silage fungal-based solid-state pretreatment for enhanced biogas production in anaerobic co-digestion with cow manure. Bioresour. Technol..

[2] Sifeeldein A., Wang S., Li J., Dong Z., Chen L., Kaka N.A., Shao T. (2019). Phylogenetic identification of lactic acid bacteria isolates and their effects on the fermentation quality of sweet sorghum (*Sorghum bicolor*) silage. J. Appl. Microbiol..

[3] Guo J., Xie Y., Yu Z., Meng G., Wu Z. (2019). Effect of *Lactobacillus plantarum* expressing multifunctional glycoside hydrolases on the characteristics of alfalfa silage. Appl. Microbiol. Biotechnol..

[4] Yu P., McKinnon J.J., Christensen D.A. (2005). Hydroxycinnamic acids and ferulic acid esterase in relation to biodegradation of complex plant cell walls. Can. J. Anim. Sci..

[5] Cao B.B., Jin X., Yang H.J., Li S.L., Jiang L.S. (2016). Microbial release of ferulic and *p*-coumaric acids from forages and their digestibility in lactating cows fed total mixed rations with different forage combinations. J. Sci. Food Agric..

[6] Cao B.B., Wang R., Bo Y.K., Bai S., Yang H.J. (2016). In situ rumen digestibility of ester-linked ferulic and *p*-coumaric acids in crop stover or straws in comparison with alfalfa and Chinese wild ryegrass hays. Anim. Feed Sci. Technol..

[7] Williamson G., Kroon P.A., Faulds C.B. (1998). Hairy plant polysaccharides: A close shave with microbial esterases. Microbiology.

[8] Soberon M.A., Cherney J.H., Liu R.H., Ross D.A., Cherney D.J.R. (2012). Free ferulic acid uptake in lactating cows. J. Dairy Sci..

[9] Crepin V.F., Faulds C.B., Connerton I.F. (2004). Functional classification of the microbial feruloyl esterases. Appl. Microbiol. Biotechnol..

[10] Xu Z., He H., Zhang S., Guo T., Kong J. (2017). Characterization of feruloyl esterases produced by the four lactobacillus species: *L*. *amylovorus*, *L*. *acidophilus*, *L*. *farciminis* and *L*. *fermentum*, isolated from ensiled corn stover. Front. Microbiol..

[11] Nsereko V.L., Smiley B.K., Rutherford W.M., Spielbauer A., Forrester K.J., Hettinger G.H., Harman E.K., Harman B.R. (2008). Influence of inoculating forage with lactic acid bacterial strains that produce ferulate esterase on ensilage and ruminal degradation of fiber. Anim. Feed Sci. Technol..

[12] Lynch J.P., Prema D., Van Hamme J.D., Church J.S., Beauchemin K.A. (2014). Fiber degradability, chemical composition and conservation characteristics of alfalfa haylage ensiled with exogenous fibrolytic enzymes and a ferulic acid esterase-producing inoculant. Can. J. Anim. Sci..

[13] Comino L., Tabacco E., Righi F., Revello-Chion A., Quarantelli A., Borreani G. (2014). Effects of an inoculant containing a *Lactobacillus buchneri* that produces ferulate-esterase on fermentation products, aerobic stability, and fibre digestibility of maize silage harvested at different stages of maturity. Anim. Feed Sci. Technol..

[14] Weinberg Z.G., Szakacs G., Ashbell G., Hen Y. (2001). The effect of temperature on the ensiling process of corn and wheat. J. Appl. Microbiol..

[15] Zhou Y., Drouin P., Lafrenière C. (2016). Effect of temperature (5–25 °C) on epiphytic lactic acid bacteria populations and fermentation of whole-plant corn silage. J. Appl. Microbiol..

[16] Bernardes T.F., Daniel J.L.P., Adesogan A.T., McAllister T.A., Drouin P., Nussio L.G., Huhtanen P., Tremblay G.F., Bélanger G., Cai Y. (2018). Silage review: Unique challenges of silages made in hot and cold regions. J. Dairy Sci..

[17] Ding Z.T., Xu D.M., Bai J., Li F.H., Adesogan A.T., Zhang P., Yuan X.J., Guo X.S. (2019). Characterization and identification of ferulic acid esterase-producing *Lactobacillus* species isolated from *Elymus nutans* silage and their application in ensiled alfalfa. J. Appl. Microbiol..

[18] Woolford M.K., Pahlow G. (1998). The silage fermentation. Microbiology of Fermented Foods.

[19] Donaghy J., Kelly P.F., McKay A.M. (1998). Detection of ferulic acid esterase production by *Bacillus* spp. and lactobacilli. Appl. Microbiol. Biotechnol..

[20] Galanis A., Kourkoutas Y., Tassou C.C., Chorianopoulos N. (2015). Detection and identification of probiotic *Lactobacillus plantarum* strains by multiplex PCR using RAPD-derived primers. Int. J. Mol. Sci..

[21] Yue Q., Yang H.J., Li D.H., Wang J.Q. (2009). A comparison of HPLC and spectrophotometrical methods to determine the activity of ferulic acid esterase in commercial enzyme products and rumen contents of steers. Anim. Feed Sci. Technol..

[22] Tian J., Li Z., Yu Z., Zhang Q., Li X. (2017). Interactive effect of inoculant and dried jujube powder on the fermentation quality and nitrogen fraction of alfalfa silage. Anim. Sci. J..

[23] Broderick G.A., Kang J.H. (1980). Automated simultaneous determination of ammonia and total amino acids in ruminal fluid and in vitro media. J. Dairy Sci..

[24] Jia T., Sun Z., Gao R., Yu Z. (2019). Lactic acid bacterial inoculant effects on the vitamin content of alfalfa and Chinese leymus silage. Asian-Australas. J. Anim. Sci..

[25] Yuan X., Wen A., Desta S.T., Wang J., Shao T. (2017). Effects of sodium diacetate on the fermentation profile, chemical composition and aerobic stability of alfalfa silage. Asian-Australas. J. Anim. Sci..

[26] Menke K.H., Raab L., Salewski A., Steingass H., Fritz D., Schneider W. (1979). The estimation of the digestibility and metabolizable energy content of ruminant feedingstuffs from the gas production when they are incubated with rumen liquor in vitro. J. Agric. Sci..

[27] Rooke J.A., Hatfield R.D. (2003). Biochemistry of Ensiling. Silage Science and Technology.

[28] Filya I., Sucu E., Karabulut A. (2004). The effect of *Propionibacterium acidipropionici*, with or without *Lactobacillus plantarum*, on the fermentation and aerobic stability of wheat, sorghum and maize silages. J. Appl. Microbiol..

[29] Alhaag H., Yuan X., Mala A., Bai J., Shao T. (2019). Fermentation characteristics of *Lactobacillus plantarum* and *Pediococcus species* isolated from sweet sorghum silage and their application as silage inoculants. Appl. Sci..

[30] Kung L., Shaver R.D., Grant R.J., Schmidt R.J. (2018). Silage review: Interpretation of chemical, microbial, and organoleptic components of silages. J. Dairy Sci..

[31] Rooke J.A., Borman A.J., Armstrong D.G. (1990). The effect of inoculation with *Lactobacillus plantarum* on fermentation in laboratory silos of herbage low in water-soluble carbohydrate. Grass Forage Sci..

[32] Wang S., Dong Z., Li J., Chen L., Shao T. (2018). Effects of storage temperature and combined microbial inoculants on fermentation end products and microbial populations of Italian ryegrass (*Lolium multiflorum* Lam.) silage. J. Appl. Microbiol..

[33] Muck R.E., Dickerson J.T. (1988). Storage temperature effects on proteolysis in alfalfa silage. Trans. ASAE.

[34] Zhang Q., Yu Z., Wang X., Tian J. (2018). Effects of inoculants and environmental temperature on fermentation quality and bacterial diversity of alfalfa silage. Anim. Sci. J..

[35] Wang M., Xu S., Wang T., Jia T., Xu Z., Wang X., Yu Z. (2018). Effect of inoculants and storage temperature on the microbial, chemical and mycotoxin composition of corn silage. Asian-Australas. J. Anim. Sci..

[36] Weinberg Z.G., Muck R.E. (1996). New trends and opportunities in the development and use of inoculants for silage. FEMS Microbiol. Rev..

[37] Petit H.V., Lafreniere C., Veira D.M. (1997). A comparision of methods to determine dry matter in silages. J. Dairy Sci..

[38] Garcia A.D., Olson W.G., Otterby D.E., Linn J.G., Hansen W.P. (1989). Effects of temperature, moisture, and aeration on fermentation of alfalfa silage. J. Dairy Sci..

[39] Li F., Ding Z., Ke W., Xu D., Zhang P., Bai J., Mudassar S., Muhammad I., Guo X. (2019). Ferulic acid esterase-producing lactic acid bacteria and cellulase pretreatments of corn stalk silage at two different temperatures: Ensiling characteristics, carbohydrates composition and enzymatic saccharification. Bioresour. Technol..

[40] Jin L., Duniere L., Lynch J.P., McAllister T.A., Baah J., Wang Y. (2015). Impact of ferulic acid esterase producing lactobacilli and fibrolytic enzymes on conservation characteristics, aerobic stability and fiber degradability of barley silage. Anim. Feed Sci. Technol..

[41] Jin L., Dunière L., Lynch J.P., Zaheer R., Turkington K., Blackshaw R.E., Lupwayi N.Z., O’Donovan J.T., Harker K.N., McAllister T. (2017). Impact of ferulic acid esterase-producing lactobacilli and fibrolytic enzymes on ensiling and digestion kinetics of mixed small-grain silage. Grass Forage Sci..

